# The transcriptome of *Leishmania major* in the axenic promastigote stage: transcript annotation and relative expression levels by RNA-seq

**DOI:** 10.1186/1471-2164-14-223

**Published:** 2013-04-04

**Authors:** Alberto Rastrojo, Fernando Carrasco-Ramiro, Diana Martín, Antonio Crespillo, Rosa M Reguera, Begoña Aguado, Jose M Requena

**Affiliations:** 1 Centro de Biología Molecular “Severo Ochoa” (CSIC-UAM), Universidad Autónoma de Madrid, 28049 Madrid, Spain; 2 Departamento de Ciencias Biomédicas, Universidad de León, 24071 León, Spain

**Keywords:** Gene Expression, RNA-seq, Transcript annotation, mRNAs, *Leishmania*, Trypanosomatids

## Abstract

**Background:**

Although the genome sequence of the protozoan parasite *Leishmania major* was determined several years ago, the knowledge of its transcriptome was incomplete, both regarding the real number of genes and their primary structure.

**Results:**

Here, we describe the first comprehensive transcriptome analysis of a parasite from the genus *Leishmania*. Using high-throughput RNA sequencing (RNA-seq), a total of 10285 transcripts were identified, of which 1884 were considered novel, as they did not match previously annotated genes. In addition, our data indicate that current annotations should be modified for many of the genes. The detailed analysis of the transcript processing sites revealed extensive heterogeneity in the spliced leader (SL) and polyadenylation addition sites. As a result, around 50% of the genes presented multiple transcripts differing in the length of the UTRs, sometimes in the order of hundreds of nucleotides. This transcript heterogeneity could provide an additional source for regulation as the different sizes of UTRs could modify RNA stability and/or influence the efficiency of RNA translation. In addition, for the first time for the *Leishmania major* promastigote stage, we are providing relative expression transcript levels.

**Conclusions:**

This study provides a concise view of the global transcriptome of the *L. major* promastigote stage, providing the basis for future comparative analysis with other development stages or other *Leishmania* species.

## Background

Species of the genus *Leishmania* are protozoan parasites and aetiological agents of a spectrum of clinical diseases, known as leishmaniases, ranging from disfiguring skin lesions to life-threatening visceral infection. The World Health Organization (WHO) estimates that 350 million people worldwide are at risk of infection, and this disease is considered a major public health problem. Two million new cases of leishmaniasis (1.5 million for cutaneous forms and 500 000 for visceral leishmaniasis) occur annually [1]. The genus *Leishmania* belongs to the order Trypanosomatida [2], which also includes, among others, *Trypanosoma brucei* and *Trypanosoma cruzi*, causative agents of two other important human infectious diseases: sleeping sickness and Chagas disease, respectively. The evolutionary origin of these organisms is found in the deepest roots of the eukaryotic tree [3], and are characterized by markedly original molecular features.

In 1999, the complete sequence of chromosome 1 of *Leishmania major* was published and showed a remarkable feature of the gene organization in *Leishmania*, i.e. genes are grouped in large clusters sharing the same transcriptional direction. Thus, from the left end of chromosome 1, the first 29 genes are all located on the same DNA strand, whereas the remaining 50 genes are located on the other strand [4]. When transcriptional activity was examined by nuclear run-on analyses using single-stranded DNA probes, the protein-coding strand was found to be more strongly transcribed than the non-coding strand in the majority of the chromosome 1 genes [5]. Furthermore, it was found that the RNA polymerase initiates transcription within the strand-switch region of chromosome 1. Similarly, in chromosome 3, which contains two convergent clusters of 67 and 30 genes, nuclear run-on analyses indicated that transcription initiates upstream of the most-5’ gene of the two long polycistronic clusters [6]. After whole genome sequences for *Leishmania* and other trypanosomatids (i.e. *T. brucei* and *T. cruzi*) were completed, it was confirmed that in these organisms most genes are organized into large clusters on the same DNA strand.

Another remarkable molecular feature found in trypanosomatids is that transcription initiation by RNA polymerase II (RNAP II) is not regulated on a per gene basis; instead, most genes are transcribed polycistronically. Genome-wide chromatin immunoprecipitation analysis of *L. major* promastigotes showed acetylated histone H3 peaks at the 5' ends of all polycistronic protein-coding gene clusters, indicating that global regulation of transcription initiation may be achieved by epigenetic regulation of H3 acetylation at the origins of polycistronic transcription units [7]. In a recent publication, the J base (a modification of thymine, which is introduced with some frequency in the DNA of trypanosomatids) was shown to define the RNAP II transcription termination sites in *L. major* and *L. tarentolae*[8].

In contrast to operons in bacteria, polycistronic units in trypanosomatids require processing before translation, and the mature mRNAs are processed from primary transcripts by coupled *trans-*splicing and polyadenylation [9]. During *trans-*splicing, a conserved spliced leader RNA (SL RNA or mini-exon) is added to the 5’ end of all mRNAs, providing the cap structure for translation. The differential expression of mature mRNAs from a single polycistronic unit is thought to be achieved by post-transcriptional control, i.e. mRNA levels are regulated by RNA stability and/or differential translation [10-12].

In 2005, the sequence for the 36 chromosomes of the *L. major* genome (32.8 Mb) was published, and provided a framework for future comparative genomic studies [13]. Using bioinformatic analyses, 911 RNA genes, 39 pseudogenes, and 8272 protein-coding genes were predicted. Within the latter group, only 36% can be assigned to a putative function based on sequence conservation with protein characterized in other eukaryotic organisms. Most *L. major* genes have orthologs in the *T. brucei* and *T. cruzi* genomes [14]. However, more than 60% of the predicted genes remain annotated as hypothetical. A major challenge lies ahead to discover whether or not these genes are expressed at any moment in the life cycle and, therefore, may be catalogued as functional genes. On the other hand, both known and putative genes lack annotated 5’ and 3’ untranslated regions (UTRs), and for only a few genes these regions have been experimentally determined [15]. In *Leishmania*, and related trypanosomatids, these flanking regions (largely the 3’-UTRs) have been involved in regulating the steady-state level and translational status of specific mRNAs along the cell cycle and in the different life cycle stages [10-12].

Recent advances in sequencing technologies, known as deep sequencing or next-generation sequencing (NGS), are becoming invaluable tools, among others, for reconstructing of the entire transcriptome of a given organism [16,17]. In this study, we employed the power of NGS on RNA analysis (RNA-seq) to provide a comprehensive characterization of the poly-A transcriptome for the promastigote stage of *Leishmania major*. A total of 10285 transcripts were identified, of which 1884 did not match with previously annotated genes and therefore were categorized as novel genes. In addition, the RNA-seq analysis generated valuable information on both the relative abundance of the RNAs and the structures of their corresponding genes (i.e. ORFs, and 5’- and 3’-UTRs).

## Methods

### *Leishmania* culture and RNA isolation

Promastigotes of *L. major* Friedlin strain (MHOM/IL/80/Friedlin; clone V1) were cultured at 26°C in RPMI medium supplemented with 10% fetal bovine serum, 100 U/ml penicillin G and 0.1 mg/mL streptomycin sulphate. Promastigotes were grown to mid log phase by seeding cultures at 1 × 10^6^ cells/mL, and collected for RNA isolation when the culture density reached 6.1 × 10^6^ cells/mL (mid-logarithmic phase of growth). Total RNA was isolated using the Aurum™ Total RNA Mini Kit (Biorad), and treated with RNAse-free DNAse I. RNA samples were quantified by absorbance at 260 nm using the Nanodrop ND-1000 (Thermo Scientific), all samples showed an A_260_/A_280_ ratio higher than 2.0. In addition, RNA integrity was checked in a bioanalyzer (Agilent 2100).

### RNA-seq and data processing

RNA-seq was performed at the Massive Sequencing Platform of Cantoblanco (CSIC-PCM, Madrid, Spain). Standard libraries for massive sequencing were generated using the TruSeq RNA Sample Prep Kit (Illumina). Briefly, poly-A^+^ RNA was selected by oligo-dT chromatography, and RNA fragmentation was achieved using divalent cations under elevated temperature. Afterwards, these fragments were used to generate a cDNA library, and cDNA fragments corresponding in size to about 300-400 bp were selected on an agarose gel. Two cDNA libraries were constructed: first strand synthesis of one of them was initiated with only random hexadeoxynucleotide primers (Illumina standard protocol); however, for the first strand synthesis of the second library, we introduced as an additional component the 5’-T_15_VN-3’ oligonucleotide together with the random hexamer primers present in the kit. Afterwards, the second strand of the cDNA was synthesized. The cDNA ends were repaired and adenylated, subsequently adapters were added at both ends. Finally, the library was enriched in ligated fragments by limited PCR amplification. Sequencing was carried out in a GAIIx Illumina system. Each library was sequenced in two separated lines. Single reads of 75 nucleotides were obtained, and raw reads were subject to quality-filtered using the standard Illumina process and analyzed using FASTQC tool [18]. Reads were mapped to the last assembled version of *L. major* genome, obtained from the Sanger Institute (ftp://ftp.sanger.ac.uk/pub/pathogens/Leishmania/major/V6_211210/), using Bowtie [19]. In the alignment of reads, a maximal of three mismatches was allowed within the whole read (aligner V mode). Nevertheless, in order to select the best alignment in terms of number of mismatches, the option “—best” was used. Also, the option “-k1” was elected, i.e. if in the course of the search Bowtie found 2 (or more) possible alignments for a given read, the program selected one of the alignments at random. We analyzed different alignment conditions in terms of multi-hits in order to obtain the best and accurate results from our data. Allowing up to 10 multi-hits for a single read, the main differences with the transcripts assembled with no multi-hits restriction were found at gene-tandem repeat regions. In those regions the assembled transcripts were reduced to the UTRs, losing the coding regions. Therefore, no restriction in the number of multi-hits was introduced, except for SL-containing reads, in which reads mapping to more than 10 sites were excluded for further analysis. Finally, mapped reads were assembled into transcripts using Cufflinks [20].

### Identification of *trans*-splicing and polyadenylation sites

Among the non-aligned reads, a search for reads containing 8 (or more) nucleotides identical to the 3’-end of the SL sequence (AACTAACGCT ATATAAGTAT CAGTTTCTGT ACTTTATTG) was performed using a custom Perl script. No mismatches were allowed. Afterwards, the SL-matching nucleotides were stripped from the reads and the remaining sequence was used to map the position of the *trans-*splicing site in the reference genome. Similarly, reads spanning potential polyadenylation sites were extracted from the non-aligned sequences by an in house Perl script, which finds reads with A-runs (higher than 5 nucleotides in length) located at an end of the read sequence. These reads were mapped back to the reference genome.

### Additional sequencing analysis tools

Samtools software [21] was used to interconvert alignment formats, and to assign the annotated genes to transcripts generated from Seqdata, a local version of Blastx program [22]. The IGV browser was used [23] for visualization of mapped reads and assembling of transcripts to its genome context. Consensus sequences were analyzed using a local version of WebLogo tool [24]. BLAST searches for sequence homologies were performed in the following databases: GeneDB [25], TritrypDB [26] and GenBank at the NCBI [27].

## Results and discussion

### RNA-seq data and delineation of transcripts

**Figure 1:**
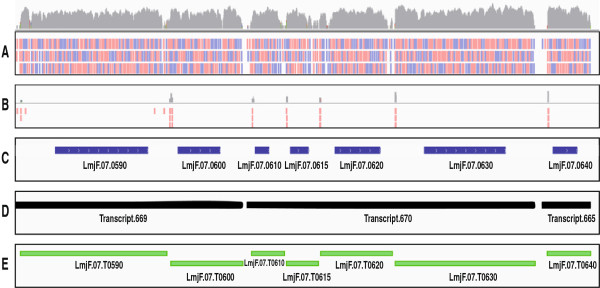
**Transcript assembling and annotation from RNASeq data.** The figure shows a region of the chromosome 7. Panel **A**: reads aligned in this region; a small window of the total mapped reads is shown (bottom panel); the relative coverage (logarithmic scale) is depicted as a sky-line on the panel. Each vertical dash represents a read. Reads aligned with the plus strand of the chromosome are shown in pink and those aligned with the minus strand in violet (note that the direction of the reads was assigned arbitrarily, as sequencing was not oriented). Panel **B**: mapping of SL-containing reads. Panel **C**: previously annotated *L. major* genome (GeneDB database). Panel **D**: crude transcripts as assembled by Cufflinks. Panel **E**: new transcript annotation after mapping of both SL addition sites and the 3’ ends generated by polyadenylation. The images were generated after loading the RNA-seq data in the Integrative Genomics Viewer (IGV 2.1) [23].

RNA isolated from an axenic culture of *L. major* promastigotes (Friedlin strain, clone V1) was sequenced, after poly(A) + selection, on an Illumina GAII platform generating a total of 14 656 121 sequence reads (75-nt long). RNA-seq data from this study have been submitted to the EBI-ENA Sequence Read Archive (SRA) under accession number ERP002077. Allowing up to three mismatches, 14 027 356 reads (95.71%) were aligned to the reference *L. major* Friedlin genome [13]. After initial assembling, it was possible to define a total of 6937 transcripts; a number lower than the 8272 protein-coding genes previously predicted to exist in the *L. major* genome [13]. However, as shown in Figure 1, this difference was not derived from a low coverage of RNA-seq data. Instead, the transcript assembly indicated that most of the *Leishmania* genome seems to be transcribed, and many assembled transcripts contain two or more annotated coding-genes (Figure 1). In fact, the genome coverage of the RNA-seq reads generated in this study was around 90.75%, even though reads for tRNAs, SL-RNAs and other small RNAs were not obtained. Several possibilities may be envisioned to accommodate this observation. First, existence of stable polycistronic transcripts; however, to date there are not descriptions of mature polycistronic transcripts in *Leishmania*. Nevertheless, the existence of a functional bicistronic transcript has been demonstrated in *T. cruzi*[28]. A second possibility is that some RNA processing intermediates with larger half-life may be represented in the RNA-seq reads. This hypothesis is very plausible as there are many reports describing processing intermediates that are clearly detected by Northern blot analysis. For example, at least 10 stable cytoplasmic poly(A) + RNAs, ranging in size from 1.7 to 13 kb and related to the 3.2-kb DHFR-TS mRNA have been observed in antifolate-resistant *Leishmania* promastigotes [29]. In other studies, polycistronic intermediates were demonstrated using a combination of genic and intergenic probes [30]. Third, antisense transcription might be contributing to create polycistronic transcript, since RNA-Seq data were derived from non-oriented, unidirectional sequencing of RNA molecules. There are several reports describing the existence of antisense transcription in *Leishmania*. For example Monnerat and co-workers [31], analyzing the transcriptional activity of a 30-Kb region from *L. major* chromosome 27, found that while the non-coding strand generally appears to be transcribed at levels close to background, several regions appeared to be transcribed at significant levels, albeit still substantially lower than the coding strand. A fourth possibility, a background derived from sequencing of contaminating DNA, may be discarded, since there are many intergenic regions from which there were no reads (see gaps without reads on Figure 1A). However, if DNA contamination were present in the RNA samples, reads should be mapped to all chromosomal locations.

In order to further delineate *Leishmania* transcripts, we took advantage of the expected addition of the 39-nucleotide long mini-exon sequence at the 5’-end of all *Leishmania* mRNAs [32,33]. Thus, we searched among the non-aligned reads (628 765; 4.29% of total reads) for sequences containing at the 5’-end eight (or more) nucleotides identical to the 3’-end of the mini-exon sequence. A total of 188 398 sequence reads met these criteria. After trimming the mini-exon sequences, these reads were aligned to the *L. major* genome (Figure 1B) and, as a result, 22 592 different mini-exon addition sites were defined.

Interestingly, only 44, of the 188 398 reads containing SL sequences, were mapped in antisense orientation (related to the coding strand), suggesting that *trans-*splicing occurs almost exclusively in sense transcripts and that antisense transcripts (if produced at meaningful levels) should not be processed by the addition of mini-exon sequences. In a recent published work [8], the authors describe the role that base J plays in termination of RNAP II transcription in *L. tarentolae*, mentioning that the vast majority of SL-containing reads were restricted to the coding strand. Proper transcription termination and avoidance of readthrough of transcriptional stops seemed to be vital for *Leishmania*[8].

As illustrated in Figure 1, most of the SL-containing reads mapped at expected locations, i.e. upstream of annotated genes and a significant number of reads were found for each putative splicing acceptor site (considering both main and alternative sites). However, exceptions for this rule were also found. Thus, from time to time, single reads containing SL sequences were mapped at unexpected positions, such as coding sequences or 3’-UTRs. Furthermore, the position of those reads was not accompanied by a breakdown in the reads density as occurs for the rest of SL addition sites. A plausible interpretation for these findings is that the *trans-*splicing machinery generates a low, but detectable number of events in which the mini-exon is misplaced. Keeping in mind this idea, we excluded in the transcript defining process those mini-exon addition sites that were defined by a sole read and located at unexpected positions.

Finally, using as criterion for defining the 5’ end of a transcript the location of a SL addition site, most of the polycistronic transcripts obtained after the initial assembling could be split up, giving a total number of 10 285 transcripts (Table 1). Only 73 of these transcripts remained as polycistronic, with 72 bicistronics and one tetracistronic (*LmjF.30.T1460-1470-1480-1490*). It would be interesting to analyze whether these bicistronic transcripts really exist or they are only evidencing current annotation deficiencies in the *L. major* database. A detailed list of the *L. major* transcriptome is provided as an Additional file 1. Transcripts were named using the systematic identifiers for the annotated genes [13], and were interdigitated numbers to name the new transcripts. In order to distinguish between transcript and genes, a T preceding the transcript number was included. By way of example, Figure 1 (panel C) shows the previously annotated genes existing in a region of chromosome 7, and in panel E are shown the new transcripts (and their names) mapped at that chromosomal region.

**Table 1:** **Transcriptome of *Leishmania major *promastigotes**

**Chromosome**	**Number of transcripts**	**Bicistronic transcripts**	**Non-annotated genes**	Mis-annotated genes (*)
1	92		7	1
2	93	2	19	9 (1)
3	110		13	9
4	140	1	11	14 (1)
5	146	1	22	4 (2)
6	154		19	7 (3)
7	158		28	6 (7)
8	171	1	36	3 (1)
9	189	1	21	6 (3)
10	175	4	32	5 (4)
11	171	1	34	1 (2)
12	183		41	12 (1)
13	207	1	38	7 (5)
14	194		35	5 (2)
15	196	2	28	11 (2)
16	213	1	37	8 (3)
17	211	1	52	5
18	242		70	5 (2)
19	216		38	4 (1)
20	215	5	40	9
21	264		37	11 (2)
22	214		45	7 (1)
23	253	3	53	8 (3)
24	286	1	48	14 (1)
25	302	4	50	22 (1)
26	330		51	10 (2)
27	340	2	59	10 (1)
28	403	1	84	13 (2)
29	372	3	77	10
30	465	2ª	69	11 (3)
31	463	5	126	51 (12)
32	509	6	85	25 (4)
33	492	5	115	13 (4)
34	570	6	88	25 (1)
35	673	5	133	9 (10)
36	873	9	143	40 (7)
**Genome**	**10285**	**73**	**1884**	**410 (94)**

*In brackets is indicated the number of genes that might be truncated by addition of SL in secondary trans-splicing sites.

^a^Transcript LmjF.30.T1460-1470-1480-1490 is tetracistronic.

With the sole exception of genes *LmjF.02.0400, LmjF.09.0690, LmjF27.0280*, *LmjF33.1760*, *LmjF35.2600* and *LmjF35.2610*, transcripts were found for all the currently annotated genes at GeneDB database [25]. These six genes code for hypotethical proteins, but, at least, gene *LmjF35.2610* seems to be encoding a protein since the predicted amino acid sequence contains a region with similarity to ubiquitin and also an AT hook, DNA-binding motif; furthermore, the gene is present in other *Leishmania* species [26]. Thus, the lack of expression of these genes, and in particular of *LmjF35.2610*, in *L. major* promastigotes is a finding that would merit further studies.

Interestingly, 1884 new transcripts were found spanning genomic regions lacking annotated genes; hence, they were categorized as non-annotated genes (Table 1). These findings suggest that the gene content of *L. major* would be approximately 20% higher than previously believed [13]. Similar results have been reported after determining the *T. brucei* transcriptome by RNA-seq [34]. Nevertheless, it is likely that many of these new transcripts may have roles other than protein-coding function; some may even be merely processing products resulting from the unusual polycistronic gene organization and processing of the *Leishmania* genome. In this regard, non-coding transcripts, derived from intercoding regions of *T. brucei* VSG genes, were found to be *trans*-spliced, polyadenylated and present in polyribosomes [35]. Therefore, the new transcripts described in this work might be considered non-coding (nc) RNAs until shown to be otherwise.

**Figure 2:**
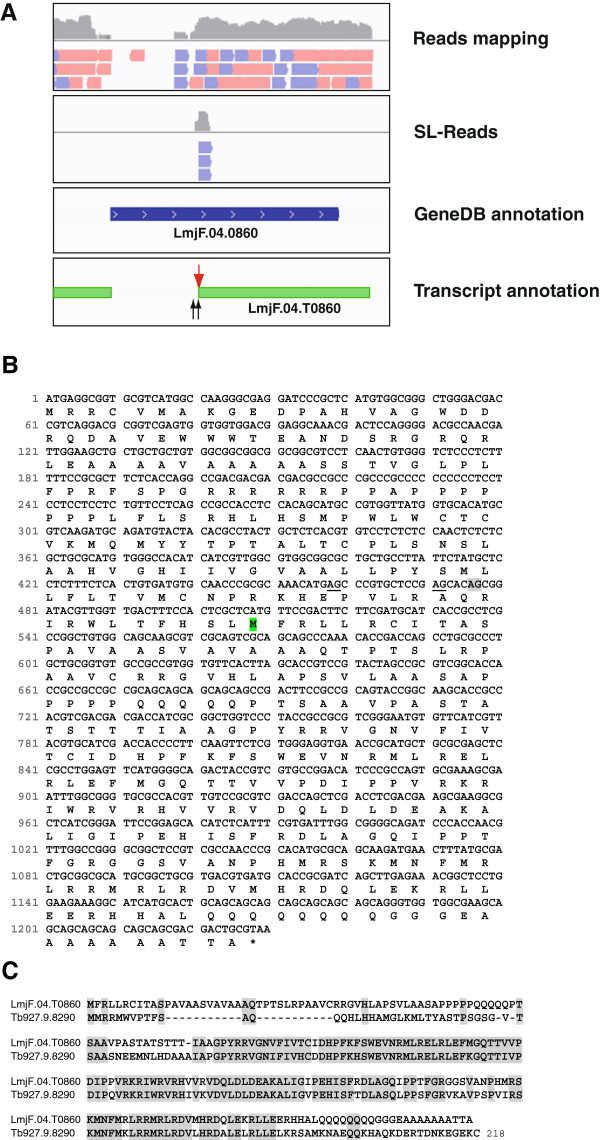
**Mis-annotation of *LmjF04.0860 *gene.** (**A**) Upper panels show the mapping of RNA-seq reads (either total or SL-containing reads) in the genomic region containing the annotated *LmjF04.0860* gene; the bottom panel contains the transcripts delimited in this region. Arrows indicate SL addition sites, the red arrow points at the main SL addition site. Reads aligned with the plus strand of the chromosome are shown in violet and those aligned with the minus strand in pink (note that the direction of the reads was a consequence of sequencing process, as sequencing was not oriented). (**B**) Nucleotide sequence (and predicted amino acid sequence) of the *LmjF04.0860* gene as annotated in the GeneDB database [25]. Shaded in gray is shown the position of the main AG dinucleotide used for *trans-*splicing in the *LmjF.04.T0860* transcripts, and underlined are those AG dinucleotide representing alternative SL addition sites. Shaded in green it is shown the first ATG found downstream the SL addition sites. (**C**) Alignment between the protein predicted in the *LmjF.04.T0860* transcript and the Tb927.9.8290 protein annotated in the *T. brucei* GeneDB database [25]. Identical amino acids are shaded in gray.

Concerning the 5’-end mapping, we have shown that 410 annotated genes are mis-predicted, regarding the translation start codons, as splice acceptor sites were found exclusively downstream of the previously assigned ATG. An example for a clear mis-annotation is shown in Figure 2. Thus, three SL addition sites were found to exist in the middle of the ORF currently annotated as *LmjF.04.0860*; however, no SL addition sites were found at the 5’ end of the annotated gene and no reads were mapped at the region coding for the N-terminal moiety of LmjF.04.0860. Translation from the nearest ATG codon found after the main SL addition site gives a protein corresponding to the last 240 amino acids of the annotated LmjF.04.0860 protein (Figure 2B). Interestingly, the new protein is similar in size and sequence to that encoded by the gene *Tb927.9.8290*, which has an authentic annotation in the *T. brucei* databases (Figure 2C). In the GeneDB database this hypothetical protein is categorized as conserved, since it is also encoded in the genomes of *T. cruzi* and other *Leishmania* species. As a structural feature, the protein contains the domain SSF55129, which is typical of the ribosomal protein L30p/L7e superfamily. In summary, these data support the conclusion that mature mRNAs containing the LmjF.04.0860 ORF, as annotated in the GeneDB database, do not exist.

On the other hand, for 94 annotated genes, alternative splice addition sites were mapped into the ORF, suggesting that different proteins might be generated from a single gene. In this regard, there is a documented case of alternative *trans*-splicing in the *T. cruzi* LYT1 gene, in which the different maturation of the mRNA leads to the expression of protein isoforms showing different compartmental and functional properties [36]. Overall, our transcriptomic study has uncovered that the current annotation of the *L. major* genome had clear limitations that are corrected by the data reported in this work.

### Determination of RNA levels from RNA-seq data

RNA-seq is an accurate method for quantifying transcript levels. The strength of this method is that it produces digital counts of transcript abundance, in contrast to the analog-style signals obtained from fluorescent dye–based microarrays. This technique has been validated by several studies and found to be highly reproducible, with very little technical variability and can measure mRNA levels over several orders of magnitude [37,38]. A useful parameter is FPKM (fragments –or reads- per kilobase of transcript per million mapped reads), which reflects the abundance of a transcript in the sample by normalizing for RNA length and for the total read number in the measurement [39]. Thus, the presence and abundance of a given RNA can be calculated and subsequently compared with the amount in any other sequenced sample, now or in the future.

Table 2 contains a list with the 50 most abundant transcripts detected in promastigotes of *L. major*. Two of the top three on the list are transcripts corresponding to the heat shock protein 70 (HSP70); this finding is not unexpected taking into account that this protein make up 2.1% of the total protein in unstressed *Leishmania* promastigotes [40]. Additionally, the fact that 20 out of 50 correspond to transcripts encoding ribosomal proteins might indicate that a direct correlation between transcript levels and protein abundance would be a general rule in *Leishmania*. Another conspicuous observation is that 18 out of the 50 most abundant transcripts derive from genes located on chromosome 35. However, at first glance, these genes do not seem to be concentrated at specific regions of the chromosome; rather they seem to be randomly distributed. Other abundant transcripts are those encoding nucleoside transporters, histone H4, peptidases, cyclophilin, LACK and tubulins (Table 2). Two abundant transcripts, *LmjF.35.T2220* and *LmjF.35.T2210*, encode KMP-11, a protein found tightly associated with lipophosphoglycan, the major cell surface glycoconjugate of *Leishmania* promastigotes [41]. In addition, there are other transcripts encoding for hypothetical proteins that are expected to be abundant ones. Thus, transcripts *LmjF.31.T0900* (the fifth on the list) would be coding for a hypothetical, small protein (79 amino acids), annotated as LmjF31.0900, which is also present in the genomes of related trypanosomatids. Interestingly, this transcript was identified in previous studies to be both abundant and differentially expressed in promastigotes by using oligonucleotide microarrays [42], and one of the most abundant transcripts in metacyclic *L. major* grown in culture by using SAGE methodologies [43]. Another abundant transcript, *LmjF.31.T0966*, located at a region lacking annotated genes contains high sequence identity with gene *LmjF31.0900*. The structural relationship between both transcripts (*LmjF.31.T0900* and *LmjF.31.T0966*) and the functional role of the encoded protein (LmjF31.0900) are two aspects that merit further studies. Transcript *LmjF.36.T3620*, containing the annotated *LmjF36.3620* gene and coding for a hypothetical protein, was also found among the most abundant transcripts in *L. major* promastigotes [42] and metacyclics forms [43].

**Table 2:** **The 50 most abundant transcripts in *L. major *promastigotes**

**Transcript**	**Gene**^**a**^	**FPKM**^**b **^**(±SD)**	**Remarks**^**c**^
LmjF.28.T2770	LmjF28.2770	1357.39 ± 5.12	heat-shock protein (HSP70; gene *HSP70-II*)
LmjF.35.T0240	LmjF35.0240	1034.68 ±12.39	ribosomal protein L30
LmjF.28.T2780	LmjF28.2780	987.24 ± 4.45	heat-shock protein hsp70 (HSP70; gene *HSP70-I*)
LmjF.36.T1940	LmjF36.1940	952.68 ± 4.37	inosine-guanosine transporter (NT2)
LmjF.31.T0900	LmjF31.0900	809.07 ± 7.12	hypothetical protein, conserved
LmjF.28.T2205	LmjF28.2205	792.33 ± 8.16	ribosomal protein S29
LmjF.35.T2220	LmjF35.2220	780.12 ± 5.99	kinetoplastid membrane protein-11 (KMP11)
LmjF.19.T0983	Non-annotated	674.85 ± 5.26	-
LmjF.35.T0600	LmjF35.0600	672.00 ± 5.81	ribosomal protein L18a
LmjF.06.T0010	LmjF06.0010	666.85 ± 8.31	histone H4
LmjF.35.T3800	LmjF35.3800	617.33 ± 7.36	ribosomal protein L23
LmjF.36.T3620	LmjF36.3620	616.48 ± 5.07	hypothetical protein, conserved
LmjF.35.T2210	LmjF35.2210	603.26 ± 4.69	kinetoplastid membrane protein-11 (KMP11)
LmjF.28.T2460	LmjF28.2460	596.61 ± 6.27	ribosomal protein S29
LmjF.20.T1285	Non-annotated	560.05 ± 5.48	-
LmjF.31.T0964	Non-annotated	539.44 ± 5.07	-
LmjF.31.T0895	Non-annotated	524.19 ± 10.51	-
LmjF.35.T3290	LmjF35.3290	514.13 ± 5.67	ribosomal protein L31
LmjF.13.T0570	LmjF13.0570	496.01 ± 5.02	ribosomal protein S12
LmjF.35.T3790	LmjF35.3790	493.33 ± 8.18	ribosomal protein L23
LmjF.35.T4191	Non-annotated	490.94 ± 5.15	-
LmjF.35.T3760	LmjF35.3760	483.03 ± 7.04	ribosomal protein L27A/L29
LmjF.30.T3340	LmjF30.3340	482.87 ± 5.89	ribosomal protein L9
LmjF.35.T2050	LmjF35.2050	464.76 ± 5.1	ribosomal protein L32
LmjF.08.T0640	LmjF08.0640	452.92 ± 3.18	hypothetical protein
LmjF.14.T0850	LmjF14.0850	451.18 ± 3.65	calpain-like cysteine peptidase
LmjF.35.T1910	LmjF35.1910	448.56 ± 5.89	ribosomal protein L15
LmjF.35.T0420	LmjF35.0420	446.58 ± 5.12	ribosomal protein S3A
LmjF.35.T1920	LmjF35.1920	446.57 ± 9.14	ribosomal protein L36
LmjF.25.T0910	LmjF25.0910	436.47 ± 3.61	cyclophilin a
LmjF.35.T3780	LmjF35.3780	427.63 ± 4.95	ribosomal protein L27A/L29
LmjF.28.T2740	LmjF28.2740	426.82 ± 4.82	activated protein kinase c receptor
LmjF.13.T0450	LmjF13.0450	425.12 ± 4.7	hypothetical protein, conserved
LmjF.20.T1280	LmjF20.1280	424.16 ± 3.22	small myristoylated protein 4
LmjF.31.T0966	Non-annotated^d^	419.44 ± 8.52	hypothetical protein, conserved
LmjF.28.T2750	LmjF28.2750	414.41 ± 4.15	activated protein kinase c receptor
LmjF.31.T1170	LmjF31.1170	414.01 ± 3.8	hypothetical protein
LmjF.35.T0410	LmjF35.0410	411.95 ± 4.52	ribosomal protein S3A
LmjF.15.T1240	LmjF15.1240	410.71 ± 3.38	nucleoside transporter 1
LmjF.24.T2230	LmjF24.2230	409.67 ± 3.11	hypothetical predicted multi-pass transmembrane protein
LmjF.35.T3280	LmjF35.3280	406.24 ± 5.44	ribosomal protein L31
LmjF.35.T0400	LmjF35.0400	403.61 ± 4.43	ribosomal protein S3A
LmjF.24.T1280	LmjF24.1280	403.46 ± 3.63	amastin-like surface protein
LmjF.13.T0370	LmjF13.0370	403.05 ± 3.84	alpha tubulin
LmjF.35.T1670	LmjF35.1670	403.05 ± 6.46	ribosomal protein L26
LmjF.13.T0360	LmjF13.0360	403.04 ± 3.84	alpha tubulin
LmjF.13.T0350	LmjF13.0350	399.2 ± 3.82	alpha tubulin
LmjF.13.T0380	LmjF13.0380	399.06 ± 3.82	alpha tubulin
LmjF.33.T3230	LmjF33.3230	396.93 ± 7.5	ribosomal protein L44
LmjF.13.T0330	LmjF13.0330	396.86 ± 3.81	alpha tubulin

^a^ GeneDB identification code.

^b^ FPKM, fragments (reads) per kilobase per million mapped reads.

^c^ Hypothetical: predicted by informatics tools; conserved: present in other trypanosomatids (i.e. *T. brucei* and *T. cruzi* genome).

^d^ This transcript has 97% of sequence identity with gene LmjF31.0900.

On the other hand, five transcripts (*LmjF.19.T0983*, *LmjF.20.T1285*, *LmjF.31.T0964*, *LmjF.31.T0895*, and *LmjF.35.T4191*), among the most abundant in *L. major* promastigotes (Table 2), do not contain previously annotated genes. The sequence of transcript *LmjF.19.T0983* was found to be conserved in the genomes of different *Leishmania* species (*L. braziliensis, L. donovani, L. mexicana* and *L. infantum*) but conserved sequences were not detected in the genomes of related trypanosomatids (i.e. *T. brucei* and *T. cruzi*). Interestingly, a cDNA (named DRS-2) derived from this transcript was previously described in *L. major as* an mRNA whose expression increases during metacyclogenesis [15]. Similarly, sequences homologous to transcript *LmjF.31.T0964* were found in the genomes of all *Leishmania* species sequenced to date, but absent in the genus *Trypanosoma*. The sequences of transcripts *LmjF.20.T1285*, *LmjF.31.T0895* and *LmjF.35.T4191* were found to be well conserved in the genomes of *L. donovani, L. mexicana* and *L. infantum*, but seemed to be absent from the *L. braziliensis* genome. It is clear that a challenge for the future will be to understand the nature (coding or not) of these transcripts, and certainly for the additional 1879 new transcripts that have been described in this work (Table 1).

**Figure 3:**
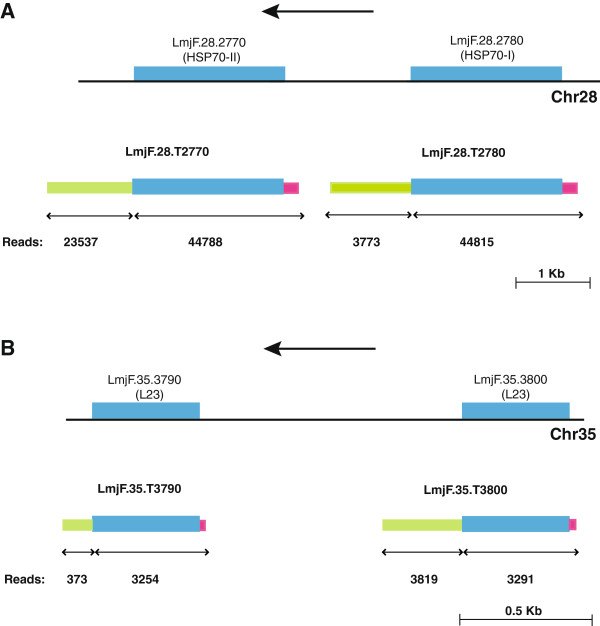
**Relative expression levels of transcripts derived from loci coding for HSP70 and ribosomal protein L23.** Panel **A**: *upper*, current annotation of the two types of genes found in the *L. major HSP70* locus, *LmjF28.2770* (also known as *HSP70-II*) and *LmjF28.2780* (*HSP70-I*); *bottom*, transcript annotation, as defined in this study, for the *L. major HSP70* locus. Panel **B**: *upper*, current annotation of the two genes coding for the ribosomal protein L23, *LmjF35.3790* and *LmjF35.3800*; *bottom*, transcripts annotated in this study. Each locus is composed by two types of genes with identical ORFs (blue boxes). Transcript mapping has allowed the identification of 5’-UTRs (purple boxes) and 3’-UTRs (green boxes). The number of reads mapped by Cufflinks within the different regions is shown at bottom.

Tandemly repeated, multi-copy gene loci are frequent in the *Leishmania* genome [11]. In fact, the list shown in Table 2 contains several examples: genes *LmjF.28.2770* and *LmjF.28.2780*, coding for HSP70; *LmjF.35.3790* and *LmjF.35.3800*, coding for ribosomal protein L23; *LmjF.35.2220* and *LmjF.35.2210*, coding for KMP-11; *LmjF35.0420*, *LmjF35.0410* and *LmjF35.0400*, coding for ribosomal protein S3A; *LmjF28.2740* and *LmjF28.2750*, coding for activated protein kinase c receptor (also known as LACK in *Leishmania*[44]); and the five *LmjF13.0330-0370* genes, coding for alpha tubulin. More frequently, the tandemly arranged genes have identical or highly conserved sequences in their protein coding regions. When RNA-seq reads are mapped at two or more places in the reference genome, due to sequence identity, the assembling algorithms, as that used in this study, make an equal distribution of the reads among the putative transcripts. Obviously, this fact may lead to miscalculation of the expression levels when two transcripts share conserved regions but also contain divergent ones. This can be illustrated analyzing the expression levels of transcripts derived from the *HSP70* locus, i.e. transcripts *LmjF.28.T2770* and *LmjF.28.T2780* (Table 2). Two types of genes, *HSP70-I* and *HSP70-II*, are present in different *Leishmania* species [45]. Both types of genes have identical 5’-UTR and coding sequences, but divergent 3’-UTRs; in addition, analysis of the steady-state mRNA levels in *L. infantum* promastigotes indicated that transcripts derived from the *HSP70-II* gene are one order of magnitude more abundant than *HSP70-I* transcripts [46]. Indeed, according to the FPKM values shown in Table 2, the level of *HSP70-II* transcripts (i.e. *LmjF.28.T2770* transcript) is higher than the level of *HSP70-I* transcripts (i.e. *LmjF.28.T2780*); however, the difference is lower than expected. Fortunately, the counter nature of the RNA-seq data allows defining with precision the transcription level of a particular region of a given gene, and Figure 3A shows the results of this analysis for the *HSP70* locus. While, as expected, the reads mapped to the 5’-UTR + CDS of both genes are equivalent, the number of reads containing sequences belonging to the 3’UTR of *HSP70-II* gene (*LmjF.28.2770*) was 6,24 fold higher than the number of reads corresponding to the 3’UTR of *HSP70-I* gene (*LmjF.28.2780*), even though the 3’-UTR lengths are very similar (1096 and 1084 nucleotides, respectively). These results indicate that the steady-state level for *LmjF.28.T2770* (*HSP70-II*) transcripts is clearly higher than that for *LmjF.28.T2780* (*HSP70-I*) transcripts, giving similar results to those determined by classical methods of mRNA expression levels [46]. Thus, this analysis demonstrated the usefulness of RNA-seq for studies of transcript abundance, and the necessity, however, of knowing and taking into account the differences in the UTR in order to determine transcript levels in a more accurate manner. To further illustrate the usefulness of RNA-seq for determining transcript abundance, we searched for another repeated genes among the more abundant transcripts listed in Table 2. We selected those coding for ribosomal protein L23 (Figure 3B). In the *L. major* genome database [25], there are two tandemly linked *L23* genes (*LmjF.35.3790 and LmjF.35.3800*). A sequence comparison showed that both genes have identical coding regions, but marked differences both in length and sequence in the 3’-UTRs. According to the number of reads obtained for each region of the genes, it was evident that transcript *LmjF.35.T3800* would be more abundant than transcript *LmjF.35.T3790* in the promastigote stage. Thus, after correcting by the length of the 3’-UTRs (93 nucleotides for gene *LmjF.35.3790* and 299 nucleotides for gene *LmjF.35.3800*), the relative steady-state level of transcript *LmjF.35.T3790* was estimated to be 3-fold lower than that for transcript *LmjF.35.T3800*.

On the other hand, our analysis evidenced a negligible level of single nucleotide polymorphisms (SNPs) in the assembled transcripts regarding the reference genome; this is a surprising discovery taking into account that *Leishmania* is an aneuploid organism, in which disomic and trisomic chromosomes are more frequently observed than monosomic ones [47,48]). Similarly, this very low rate of heterozygosity was noted when sequencing the *L. major* genome [13] and, more recently, when Rogers and co-workers re-sequenced the *L. major* genome using the Illumina methodology [48].

### Heterogeneity of *trans*-splicing and polyadenylation sites

**Figure 4:**
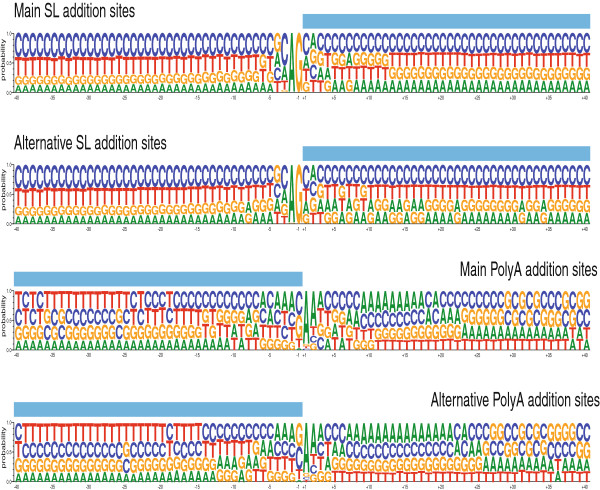
**Nucleotide frequencies for sequences surrounding SL and polyadenylation addition sites.** Panels show the compositional profiles of sequences around the main SL addition sites (n = 9530), alternative SL addition sites (n = 4531), main polyadenylation sites (n = 3178) and alternative polyadenylation sites (n = 1238).

The addition of a 39-nt mini-exon (or spliced leader, SL) to the 5’ end of all mRNAs in *Leishmania* and related trypanosomatids provides the 5’ cap structure for mRNA translation [32]. As noted above, we have obtained a large number of mini-exon-containing reads and this facilitated the 5’ end-mapping for most transcripts. Furthermore, we mapped two or more SL addition sites for around 50% of the genes, suggesting the existence of a remarkable heterogeneity in the selection of the SL addition site. A similar observation has been reported in RNA-seq studies carried out in a related trypanosmatid, *T. brucei*[34,49,50]. For bioinformatics analyses, when the distance between two consecutive SL addition sites was lower than 500 nucleotides, they were considered alternative addition sites for a given gene. Furthermore, taking into account the numbers of reads mapping at each site, they were categorized as either main addition sites or alternative splicing sites. Thus, SL addition sites were separated into two categories: i) main SL addition sites, including unique SL addition sites or the most frequent SL addition sites when two or more sites were mapped in the same transcript; ii) alternative SL addition sites, i.e. the rest of SL addition sites in transcripts containing two of more SL addition sites. In order to avoid possible bias, SL-containing reads mapping to ten or more different genomic positions were excluded from this analysis. Most of these “multi-hit” reads mapped to the 5’-UTR of gene families containing ten or more members (Crespillo et al, manuscript in preparation). Finally, a total of 9530 SL addition sites were classified as main sites and 4531 as alternative ones. Looking for sequence signatures associated with the SL addition sites, a compositional analysis in the ± 40 nucleotide region surrounding the SL addition site was carried out (Figure 4). Addition of SL occurs after the well-known AG dinucleotide, even though a slight difference was observed between main and alternative sites. Thus, whereas for the main SL addition sites, the A and G frequencies were 99.82% and 99.87%, respectively, for the alternative sites the A and G frequencies were 97.26 and 99.23%. Additionally, in agreement with previous analysis [51], a preference for a C before the AG dinucleotide was observed. Again, this preference was more marked in the main addition sites (55.26%) than in the alternative addition sites (40.87%). Another noticeable feature of the sequence surrounding the AG addition site is a clear richness in pyrimidine nucleotides (Figure 4). This T + C richness is more pronounced in the upstream region: in the -40 to -21 positions the T + C frequencies are higher than 70%. Likewise, the T + C content is higher in the regions upstream the main addition sites (71.28%) than the alternative ones (68.68%).

Heterogeneity in the polyadenylation sites in the *Leishmania* transcripts was also observed; however, the number of reads found denoting polyadenylation events was lower (7894 reads) than those mapped at the 5’end (see above), in spite that we prepared a second library in which an oligo-dT for priming was included in the cDNA reaction (see Methods section). Difficulties in the identification of polyadenylation sites were also experienced by other authors [34]. Recently, P.J. Myler and coworkers have deposited in the TriTrypDB database [26] a large number of SL- and polyadenylation sites for *L. major*; these new data further illustrate the complexity of *trans-*splicing and polyadenylation site selection in *Leishmania*. A comparative study between our data and those from Myler’s laboratory is underway. Nevertheless, some conclusions may be drawn from the analysis of those reads mapping at the polyadenylation sites derived from our data. Polyadenylation sites were categorized as main (3178 different sites) and alternative (1238 sites). A compositional analysis of the regions surrounding the polyadenylation sites for both categories is shown in Figure 4. Searching for possible consensus sequence, we followed the consensus criteria defined by Cavener [52]: a consensus status is assigned to a single base when the frequency of a nucleotide at a certain position is greater than 50% and greater than twice the relative frequency of the second most frequent nucleotide; a pair of bases were assigned co-consensus status if the sum of the relative frequencies of the two nucleotides exceeded 75%. The application of this rule leads to a very short consensus for the polyadenylation addition site, which may be defined as (C/G)AA; the noteworthy differences between main and alternative sites were: i) C is more frequent in the main sites (40.62%) than in the alternative ones (38.77%); ii) the frequencies for A residues at position 2 and 3 are higher in the main sites (88.92 and 59.1%, respectively) than those found in the alternative sites (83.84 and 51.93%, respectively). An unresolved question related to the polyadenylation consensus sequence is whether the polyadenylation occurs either before or after the AA dinucleotide. Although our data cannot elucidated this question, it is likely that the adenosines of the consensus sequences are encoded residues as it is well known that poly(A) polymerases prefer an initial adenosine residue for attachment of the poly(A) tail, and therefore the selection of the polyadenylation site would be strengthened by the presence of adenosine residues [53].

## Conclusions

Sequencing and annotation of the genomes for some *Leishmania* species [13,54] have constituted an important milestone for the study of many biological aspects of this group of parasites. The availability of these genome sequences [25,26] now enables database mining and identification of different protein sets in *Leishmania*. This information provided new approaches to study the pattern of gene expression during differentiation and development by the use of DNA microarrays [55]. In current genome databases, the *Leishmania* genes lacks the definition of 5’ and 3’ UTRs; however, it should be noticed that recently P.J. Myler and coworkers have incorporated SL and polyA sites for most genes of *L. major* in the TriTrypDB datase [26]. The RNA-seq study described here represents the first annotation of the *L. major* transcriptome, in which the genes have been delimited in their translated and untranslated regions. As a result, we have uncovered many cases of mis-annotated genes, and more importantly we have found 1884 new genes (previously non-annotated) in the promastigote stage. In addition, we have determined relative expression levels for each one of the 10285 transcripts detected in *L. major* promastigotes. In summary, the data generated by this study constitute a framework for future analysis aimed to determine differential gene expression either along the life cycle or among different *Leishmania* species.

## Competing interests

The authors declare that they have no competing interests.

## Authors’ contributions

FC, BA and JMR were responsible for design and coordination of this study. AR carried out most of the bioinformatics analyses; DM, AC, RMR and JMR contributed to data analysis. JMR and BA wrote the manuscript. All the authors read and approved the final manuscript.

## Supplementary Material

Additional file 1 **An Excel file containing the complete transcriptome information for each one of the 36 *L. major *chromosomes.** The coordinates for each transcript are provided (Locus column), in addition to location of main (SL) and alternative SL (Alt_SL) addition sites, location of main (polyA) and alternative (Alt_polyA) polyadenylation sites. It is also indicated (GeneDB_ID) whether the transcript corresponds to a previously annotated gene (in this case the corresponding annotated gene is provided) or represents a new gene (denoted as unknown). The relative levels, expressed as FPKM, for each transcript are also provided. Finally, transcripts evidencing mis-annotated translation start codons are remarked as truncated. Click here for file
